# Fatty acid accumulation in feeding types of a natural freshwater fish population

**DOI:** 10.1007/s00442-021-04913-y

**Published:** 2021-04-25

**Authors:** Kristin Scharnweber, Fernando Chaguaceda, Peter Eklöv

**Affiliations:** 1 Department of Ecology and Genetics, Limnology, Uppsala University, Uppsala, Sweden; 2 Present Address: Plant Ecology and Nature Conservation, University of Potsdam, Potsdam, Germany; 3 Present Address: Department of Aquatic Sciences and Assessment, Swedish University of Agricultural Sciences, Uppsala, Sweden

**Keywords:** Fatty acid conversion, Compound-specific stable isotope analysis, Docosahexaenoic acid, Bioconversion, Trophic upgrading

## Abstract

Fatty acids are widely used to study trophic interactions in food web assemblages. Generally, it is assumed that there is a very small modification of fatty acids from one trophic step to another, making them suitable as trophic biomarkers. However, recent literature provides evidence that many fishes possess genes encoding enzymes with a role in bioconversion, thus the capability for bioconversion might be more widespread than previously assumed. Nonetheless, empirical evidence for biosynthesis occurring in natural populations remains scarce. In this study, we investigated different feeding types of perch (*Perca fluviatilis*) that are specialized on specific resources with different levels of highly unsaturated fatty acids (HUFAs), and analyzed the change between HUFA proportions in perch muscle tissue compared to their resources. Perch showed matching levels to their resources for EPA, but ARA and especially DHA were accumulated. Compound-specific stable isotope analyses helped us to identify the origin of HUFA carbon. Our results suggest that perch obtain a substantial amount of DHA via bioconversion when feeding on DHA-poor benthic resources. Thus, our data indicate the capability of bioconversion of HUFAs in a natural freshwater fish population.

**Supplementary Information:**

The online version contains supplementary material available at 10.1007/s00442-021-04913-y.

## Introduction

Besides the important macronutrients (i.e. carbon, nitrogen, and phosphorus), consumers are highly reliant on complex organic compounds, such as polyunsaturated fatty acids (PUFAs). These biochemical compounds are parts of lipids and play major functional and structural roles in cell membranes, and many other physiological processes, for instance, in hormone release, disease susceptibility and immune responses (Parrish 2009). Highly unsaturated fatty acids (HUFAs) with 20 or more carbon atoms from the n-3 or n-6 family [for example, arachidonic acid (ARA), 20:4n-6; eicosapentaenoic acid (EPA), 20:5n-3; or docosahexaenoic acid (DHA), 22:6n-3] are especially important in organism physiology. They have been linked to increased production in consumers (Kainz et al. 2004; Twining et al. 2016). Furthermore, evidence is accumulating that individuals feeding on a HUFA-rich diet have an increased fitness, for instance, a higher reproductive output (Twining et al. 2018) or an increment of immune functions (Fritz et al. 2017), compared to their conspecifics feeding on a HUFA-poor diet. In contrast, dietary HUFA limitation can result in severe detrimental effects, including decreased growth rate and impaired sensory abilities (Brenna et al. 2014; Twining et al. 2016).

Generally, we find that dietary fatty acid composition is reflected in the consumer’s tissue (Iverson 2009; Tocher 2003). This has led to the development of fatty acids being used as trophic biomarkers to illustrate trophic relationships (Napolitano 1999; Scharnweber et al. 2016). Therefore, many ecological studies of fatty acids assume that the PUFA content in consumers remains unchanged compared to the one of their resources. However, this view ignores the ability of consumer to modify fatty acids via internal transformation, such as selective retention or mobilization, and bioconversion, by elongating and/or desaturating shorter chained fatty acid precursors into HUFAs (Bell et al. 2009; Twining et al. 2016). To understand the ability and the extent of consumers to modify the dietary fatty acids is of critical importance for quantitative and qualitative approaches of fatty acids in ecological research (Galloway et al. 2020). The major pathways for the conversion of HUFAs (i.e. the derivation of these compounds from shorter chain precursors) occur either from α-linolenic acid (ALA; 18:3n-3) via EPA to DHA or from Linoleic acid (LIN; 18:2n-6) to ARA (Bell et al. 2009; Monroig et al. 2013). Such trophic upgrading was shown for several fish species (Monroig et al. 2013). Generally, freshwater fish species have a higher ability to transform dietary precursors to HUFAs compared to marine species to compensate for the low availability and abundance of n-3 HUFAs, especially DHA in freshwater habitats (Sargent et al. 1999). For example, Ishikawa et al. (2019) showed a relationship between the duplication of the key enzyme *Fads2* catalyzing the desaturation in DHA biosynthesis and the colonization of freshwater habitats in several ray-finned fish species. This study reported the molecular basis of the enzyme activities, thus, the presence and expression of candidate genes responsible for the syntheses of the key enzymes necessary for trophic upgrading of HUFAs. However, the efficiency and the extent of these routes in a natural population is unknown. Evidence of HUFA bioconversion in fish exist primarily from feeding trials in aquaculture (e.g. Katan et al. 2019; Murray et al. 2014; Xu et al. 2002), while studies on the capability and ubiquity of bioconversion under natural conditions are scarce.

In previous studies, we found that Eurasian perch (*Perca fluviatilis*) specializing on benthic invertebrates, a resource low in DHA, showed similar proportions of DHA in muscle tissue compared to perch feeding primarily on zooplankton of high-DHA content (Scharnweber et al. 2016). Along the same line, Chaguaceda et al. (2020) found that 72% of the fatty acid variation in perch remained unexplained by diet, suggesting that other factors, for example, bioconversion may also affect the fatty acid composition.

One major challenge is to understand the effects of variable levels of HUFAs for the individual diet composition in natural omnivorous populations. Stable isotope analyses in conjunction with isotope mixing models provide an excellent tool for this purpose, as the values of *δ*^13^C and *δ*^15^N of the consumers in comparison to their potential prey can help to characterize the specific composition of the diet (e.g. Boecklen et al. 2011).

Here, we tested the relationship between different resource HUFA supplies occurring on the natural scale and the individual specialization in the common fish predator perch. Individual specialization in perch is well-studied and in many Swedish lakes, different feeding types of perch exist with strong individual preferences for zooplankton, benthic invertebrates or fish resources, followed by morphological adaptions (e.g. Scharnweber et al. 2016; Svanbäck et al. 2002, 2003; Svanbäck et al. 2015). Further, we compared the levels of HUFAs of the different feeding types with the ones of their resources by extending a previous dataset on fatty acid variation of specialized perch in Lake Erken, Sweden (Chaguaceda et al. 2020). Thus, we estimated the accumulation of fatty acids from resources to consumers sensu Kainz et al. (2004). We focused on three specific HUFAs that are of high physiological importance: ARA, EPA, and DHA, and investigated the degree of internal bioconversion in the different feeding types. While perch individuals usually show a preference for specific dietary items, they still feed on a mix of resources. For example, perch assigned as benthic feeders, ingesting primarily low-quality invertebrates, still have a variable degree of high-quality zooplankton in their diet (Scharnweber et al. 2016; Svanbäck et al. 2015). The question arises whether this small amount would be sufficient to fulfill HUFA requirements, or whether HUFAs need to be converted from shorter chain PUFAs. To obtain specific information on the origin of HUFAs, compound-specific isotopic analyses (CSIA) provides a valuable tool (Twining et al. 2020). In previous studies, this approach helped to trace whether specific HUFAs have a common or distinct carbon sources (Chamberlain et al. 2004; Katan et al. 2019; Koussoroplis et al. 2010). In bulk stable isotope samples, a general difference between benthic and pelagic primary producers exist, with benthic producers being enriched in ^13^C, leading to more positive values (France 1995). Potentially, this pattern will also be visible in the δ^13^C of HUFAs, and could help to identify the carbon habitat of origin as well as giving indications of bioconversion.

The objectives of our study were twofold. First, we investigated the accumulation of different physiologically important HUFAs in perch feeding types, i.e. we compared the realized HUFA levels in perch muscle tissue to the ones that are theoretically assumed from diet. We did this by obtaining diet contributions from stable isotope mixing models to detect whether there is a match or mismatch in the proportions of HUFAs between resources and consumers. Second, we applied CSIA to find indications for bioconversion in perch. We hypothesize that feeding types with a low-HUFA diet would exceed the theoretically assumed HUFA levels from diet, but *δ*^13^C of the HUFA carbon would still reflect the carbon in resources from the specific feeding habitat.

## Methods

In August 2015, we sampled perch from Lake Erken (59°50′09.6″N, 18°37′52.3″E) in Central Sweden which has been extensively studied with respect to dietary specialization (e.g. Chaguaceda et al. 2020; Marklund et al. 2019; Svanbäck et al. 2003). Perch and potential prey fish (ruffe, *Gymnocephalus cernua* and roach, *Rutilus rutilus*) were caught using multimesh gill nets (littoral nets: 30 × 1.5 m; pelagic nets: 27.5 × 6 m) that were set overnight, either in the shallow near-shore littoral zone, or the open-water pelagic zone. After measuring and weighing, the fish were frozen to − 20 °C. In the lab at Uppsala University, they were partially thawed and fish muscle tissue samples were taken for stable isotope analyses (δ^13^C and δ^15^N), fatty acid analyses, and compound-specific stable isotope analyses of fatty acids.

Zooplankton and macroinvertebrate resource samples were collected during the fishing campaign to be used for stable isotope analysis. Zooplankton samples were obtained by hauling a 100 µm net through the whole water column to obtain sufficient biomass, whereas macroinvertebrates were caught with a sweep net and picked from stones and plants in the littoral zone. After allowing for gut clearance and separation into the major taxonomic groups, they were, together with the fish stable isotope samples, dried in an oven for 24 h at 60 °C.

Values for proportions of HUFAs in perch invertebrate resources (i.e. Cladocera, Copepoda, and benthic macroinvertebrates, consisting of Chironomidae, Trichoptera, Isopoda, and Ephemeroptera) were taken from a previous study that collected perch resources in Lake Erken and two other similar lakes in Central Sweden (Scharnweber et al. 2016). We assume that these samples reflect the quality of food sources for the fishes in this study.

### Sample analyses

#### Stable isotope, mixing model, and clustering analyses

Stable isotope analyses were conducted as described in Chaguaceda et al. (2020). Samples were ground using a mortar and pestle and approximately 1 mg was weighed and transferred into tin capsules that were sent to the Stable Isotope Facility at the University of California, Davis, California, USA for analyses of δ^13^C and δ^15^N using a PDZ Europa ANCA-GSL elemental analyzer coupled to a PDZ Europa 20–20 isotope ratio mass spectrometer (Sercon, Cheshire, UK).

Feeding types of perch were assigned based on the dietary contributions from Bayesian mixing models conducted in MixSIAR (version 3.1.10) (Stock et al. 2018) in R (R Core Team 2018). Prior to conducting mixing models, the isotope dataset was divided into perch relying on pelagic and benthic pathways, respectively, using k-means clustering (Clarke et al. 2014). Mixing models were calculated separately for each of the clusters and the structure was hierarchical with perch individual nested within age, a general prior distribution and process error structure. Age data were obtained from opercular bones (Le Cren 1947) and length-at-age was assessed from regressions of total length and opercular diameter for littoral and pelagic perch separately (Francis 1990).

Copepoda, Cladocera, benthic macroinvertebrates (consisting of Chironomidae, Gastropoda, and Isopoda), and prey fish (ruffe and roach) were used as endmembers in the mixing models for all 113 perch individuals and trophic fractionation factor was set at 0.4 ± 1.3‰ for δ^13^C and 3.4 ± 1.0‰ for δ^15^N (Post 2002). Pelagic contribution was calculated by summing the contributions of Copepoda and Cladocera.

For obtaining feeding types based on diet and habitat, k-means clustering was conducted on mixing model outputs, resulting in five feeding types of perch: littoral benthivorous, littoral planktivorous, pelagic benthivorous, pelagic planktivorous, and littoral piscivorous perch (Online Resource 1).

### Fatty acid analyses

We analyzed fatty acids following the approach described in Scharnweber et al. (2016) and Chaguaceda et al. (2020). At Uppsala University, lipids were extracted from muscle tissue of fish in a solution of chloroform/methanol (2:1 by volume), and 0.88% potassium chloride in water was added to remove the non-lipids. Extraction procedure was repeated and sonification (10 min) was used to enhance extraction. Extracts were concentrated under a nitrogen stream, dissolved in hexane and transmethylated at 90 °C for 90 min. using 1% H_2_SO_4_ in methanol as a catalyst. Fatty acid methyl esters (FAMEs) were analyzed using an Agilent 6890N Gas Chromatographer with a Agilent MSD 5977A single quadrupole mass selective detector (Agilent Technologies, Santa Clara, USA) equipped with a DB-23 column (length 30 m, ID 0.25 mm, film thickness 0.25 μm; Agilent). Samples were injected in split mode with helium as a carrier gas with an average flow rate of 0.8 ml min^−1^ (initial temperature 180 °C, increase by 2 °C min^–1^ until 210 °C with 2 min hold). FAME peaks were identified using retention times and mass spectra, and a heneicosanoic acid (Nu‐Chek Prep, Elysian, Minnesota, USA) was used as an internal standard. Fatty acid concentrations were calculated using the calibration curves based on the standard solutions of known lipid mixtures (Nu‐Chek Prep). We identified 38 fatty acids but in this study focused on ARA, EPA, and DHA only.

### CSIA

*δ*^13^C of perch fatty acid methyl esters (FAMEs) were analyzed using a Trace 1310 GC gas chromatograph, equipped with a BPX-70 column (60 m, ID 0.25 mm, film thickness 0.25 μm) (Trajan Scientific Australia, Ringwood, Australia) coupled to a Thermo MAT 253 IRMS through a GC IsoLink II combustion interface (Thermo Fisher Scientific, Waltham, MA, USA).

The samples were introduced with a splitless injection at an initial temperature of 250 °C for 1 min (constant flow rate 1.4 mL min^−1^), and the program was set for 80 °C (hold 1 min), then increasing by 3 °C min^−1^ until reaching 210 °C (hold 5 min), finally increasing by 3 °C min^−1^ until reaching 245 °C (hold 5 min). *δ*^13^C values were corrected using reference mixtures, composed of pure FAMEs of calibrated *δ*^13^C. The *δ*^13^C of individual HUFAs were calculated by correcting for methyl C atoms added during derivatization according to the formula:

$$\delta^{13} {\text{C}}_{{{\text{HUFA}}}} = \frac{{(n + 1 \times \delta^{13} {\text{C}}_{{{\text{FAME}}}} - 1 \times \delta^{13} {\text{C}}_{{{\text{Methanol}}}} )}}{n},$$

where *n* is the number of C atoms in the HUFA, $\delta^{13} {\text{C}}_{{{\text{FAME}}}}$ is the isotope ratio of the measured FAME and $\delta^{13} {\text{C}}_{{{\text{Methanol}}}}$ is the isotopic ratio of the used methanol.

### Statistical analyses

HUFA proportions of perch resources were compared. Thus, roach and ruffe collected in this study were taken as a proxy for resource quality of fish prey, while proportions of Cladocera, Copepoda, and benthic invertebrates (Chironomidae, Trichoptera, Isopoda, and Ephemeroptera) were taken from Scharnweber et al. (2016). Differences in proportions of ARA, EPA, and DHA between the different resources were tested using a Kruskal–Wallis test with Bonferroni-adjusted Dunn’s pairwise comparisons.

Similar to the approach described by Hessen et al. (2006), we calculated accumulation factors for the perch individuals to compare the theoretical expected HUFA proportions (based on diet) with the realized proportions in perch muscle tissue. To obtain a proxy for the amount of HUFAs perch will receive by feeding on specific resources, we standardized the average concentrations (measured as µg fatty acids mg DW^−1^) of ARA, EPA, and DHA in the perch resources to the total mass of fatty acids quantified, as recommended by Happel et al. (2017). Then we multiplied these obtained PUFA proportions by the average dietary proportions obtained from the MixSIAR models. Pelagic contributions consisted of a mixed diet of Cladocera and Copepoda. As these resources have very different HUFA contents, we weighted the contribution from both resources according to average resource use values obtained from stomach content in Erken perch (Marklund et al. 2019). By comparing the accumulation factors in the different feeding types of perch, we were able to test if muscle tissue in perch would simply reflect the HUFA content of their resources. Any significant divergence from this hypothesized relationship would indicate selective retention. If the accumulation factor would be 1, the HUFA proportions between resources and perch muscle tissue would match. Values above 1 would translate into higher proportions of HUFAs in perch muscle tissue than expected by the proportions found in their food, and would indicate accumulation and potentially bioconversion.

We tested if the accumulation factor for ARA, EPA, and DHA differs between the feeding types by applying a Kruskal–Wallis test with Bonferroni-adjusted Dunn’s pairwise comparisons.

Differences in HUFA *δ*^13^C between the feeding types were explored using a Kruskal–Wallis test with Bonferroni-adjusted Dunn’s pairwise comparisons.

We used IBM SPSS (version 25) for frequentist statistics. The study was approved by the Uppsala Animal Ethic Committee with permit number: 267 C59/15.

## Results

We base our study on 113 individuals of perch, of which 12 were assigned as littoral benthivorous, 28 as littoral planktivorous, 2 as pelagic benthivorous, 55 as pelagic planktivorous, and 16 perch individuals were assigned as littoral piscivorous perch (Online Resource 1).

### HUFA content of resources

**Fig. 1:**
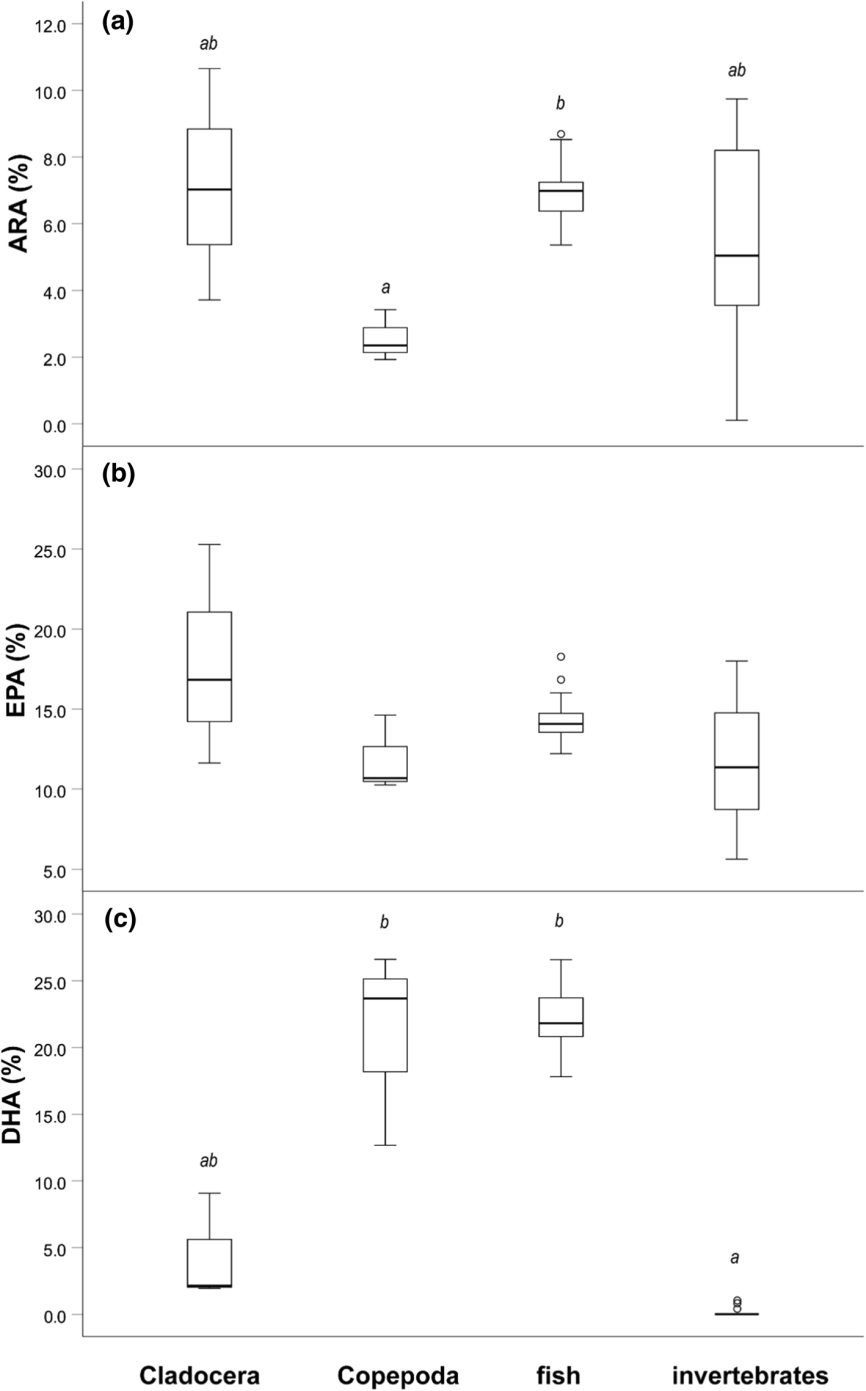
Proportions of HUFAs in perch resources. Boxplots depict resource proportions for **a** ARA; **b** EPA; and **c** DHA of zooplankton [Cladocera (*N* = 3) and Copepoda (*N* = 3)), fish (*N* = 4), and benthic invertebrates (*N* = 14)]. Feeding types with the same letter are not significantly different (Bonferroni-adjusted Dunn’s pairwise comparisons). Boxplots depict median, 25th and 75th percentile, and whiskers extend to maximum and minimum values, except for outliers (> 1.5 times box height, represented by dots)

In general, perch resources varied significantly in proportions of ARA, (Kruskal–Wallis: *H* = 12.200, *df* = 3, *P* = 0.007), EPA (Kruskal–Wallis: *H* = 7.806, *df* = 3, *P* = 0.050), and DHA (Kruskal–Wallis: *H* = 40.878, *df* = 3, *P* > 0.001). Cladocera (average 7.1% ± 3.5% standard deviation, SD) and fish resources (6.9% ± 0.7 SD) contained high proportions of ARA, whereas Copopoda (2.6% ± 0.8 SD) were lower and invertebrates (5.5% ± 3.0 SD) showed a high variation in ARA (Fig. 1a). Pairwise comparisons depicted significant differences between ARA proportions of Copepoda and fish (Fig. 1a). Cladocera showed highest proportions of EPA (17.9% ± 6.9 SD), followed by fish (14.2% ± 1.0 SD), whereas invertebrate resources were characterized by lower proportions (11.5% ± 3.8 SD) (Fig. 1b). However, Bonferroni-adjusted Dunn’s pairwise comparisons did not depict any significant difference. DHA proportions were found highest in fish (22.2 ± 2.0% SD) and Copepoda (21.0 ± 7.3% SD), whereas Cladocera showed lower proportions (4.4 ± 4.1% SD), and invertebrates even lower proportions 1% (0.2 ± 0.5% SD) (Fig. 1c). Pairwise comparisons in DHA proportions were significantly different between invertebrates and fish, and invertebrates and Copepoda (Fig. 1c). These results are in-line with previous studies comparing differences between these taxa in natural populations (Lau et al. 2012; Persson and Vrede 2006; Strandberg et al. 2015).

### Relationships between realized and theoretical HUFA proportions in perch

**Fig. 2:**
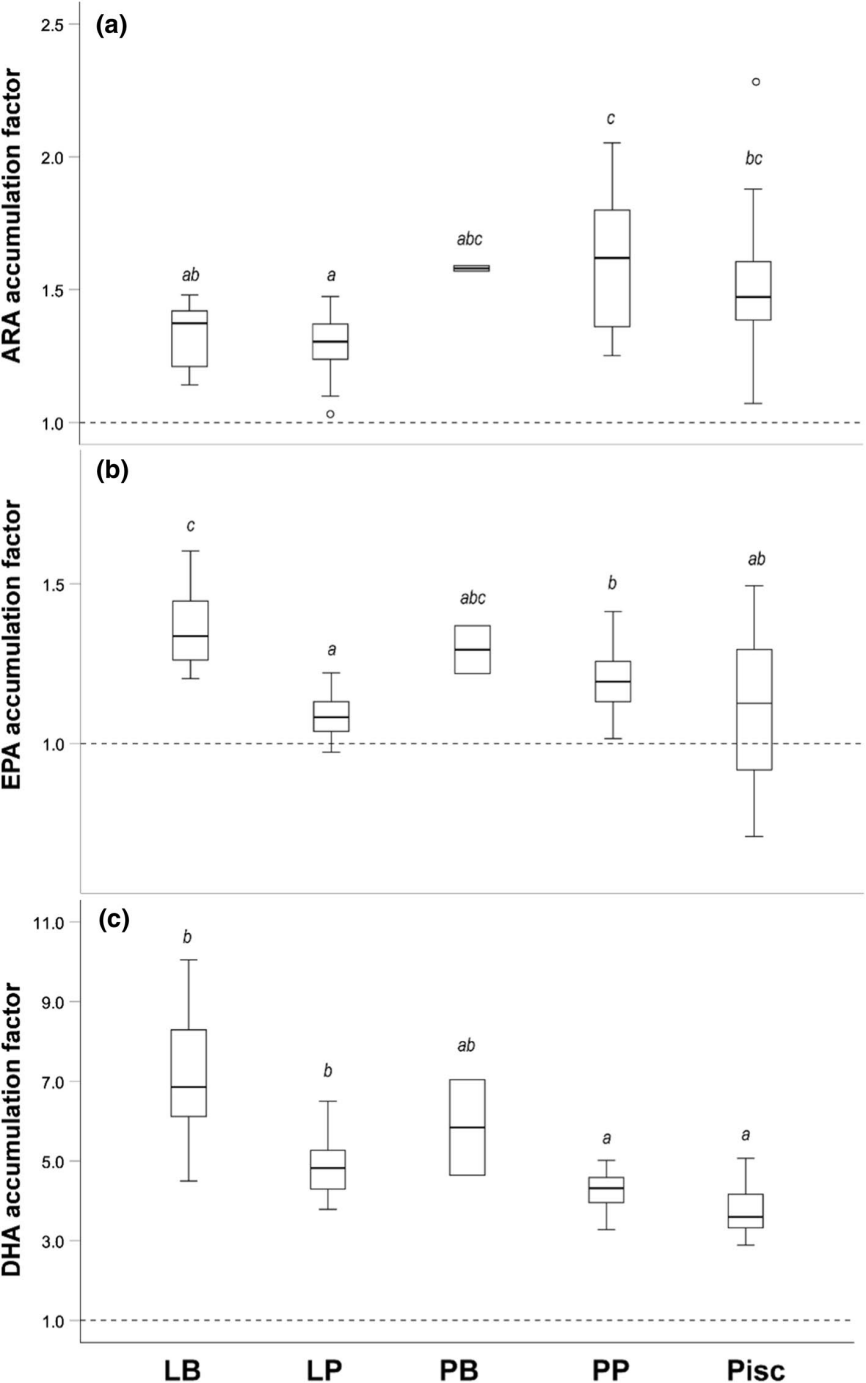
Accumulation factors for the different feeding types in perch. Boxplot are depicted for **a** ARA; **b** EPA; **c** DHA in littoral benthivorous (LB), littoral planktivorous (LP), pelagic benthivorous (PB), pelagic planktivorous (PP), and littoral piscivorous perch (Pisc). Factors close to 1 (depicted by dashed line) indicate a match between the proportions of resources and perch muscle tissue, whereas factors above 1 would be translated to higher proportions of HUFAs in perch muscle tissue than expected by the proportions found in their food, indicating accumulation. Feeding types with the same letter are not significantly different (Bonferroni-adjusted Dunn’s pairwise comparisons). Boxplots depict median, 25th and 75th percentile, and whiskers extend to maximum and minimum values, except for outliers (> 1.5 times box height, represented by dots)

The accumulation factor of ARA showed significant differences between the feeding types (Kruskal–Wallis: *H* = 34.16, *df* = 4, *P* < 0.001) and varied around an average of 1.48 (± 0.25 SD) (Fig. 2a). Factors were lowest in littoral planktivorous perch (1.30 ± 0.11 SD, significant differences to factors of pelagic planktivorous and piscivorous perch) and highest in piscivorous (1.51 ± 0.28 SD) and pelagic planktivorous perch (1.60 ± 0.25 SD) (Fig. 2a).

Similarly, the accumulation factor of EPA differed significantly between the feeding types (Kruskal–Wallis: *H* = 40.22, *df* = 4, *P* < 0.001) and varied closer to 1 (1.18 ± 0.14 SD), indicating matching proportions between resources and perch. Factors were lowest in littoral planktivorous perch (1.08 ± 0.06 SD) and piscivorous perch (1.12 ± 0.24 SD), and highest in littoral benthivorous perch (1.37 ± 0.14 SD) (Fig. 2b). Post hoc comparisons revealed significant differences of littoral benthivorous perch to all other feeding types.

Furthermore, the accumulation factors of DHA differed significantly between feeding types (Kruskal–Wallis: *H* = 53.62, *df* = 4, *P* < 0.001). However, for this HUFA, factors were greater than 1 (average 4.67 ± 1.20 SD), indicating almost five times higher DHA proportions in muscle tissue compared to the assumed proportions based on dietary contribution (Fig. 2c). Significant differences were found between the lowest factors of piscivorous perch (3.70 ± 0.57 SD) and the highest in littoral benthivorous perch (7.15 ± 1.56 SD) (Fig. 2c).

### Bulk SIA of resources and CSIA of perch fatty acids

Bulk δ^13^C of pelagic zooplankton, consisting of Cladocera and Copepoda was on average − 29.51 ± 1.63‰ SD. In contrast, δ^13^C of benthic invertebrates, consisting of Chironomidae, Gastropoda, and Isopoda, was on average − 22.35 ± 0.90‰ SD (Online Resource 1).

**Fig. 3:**
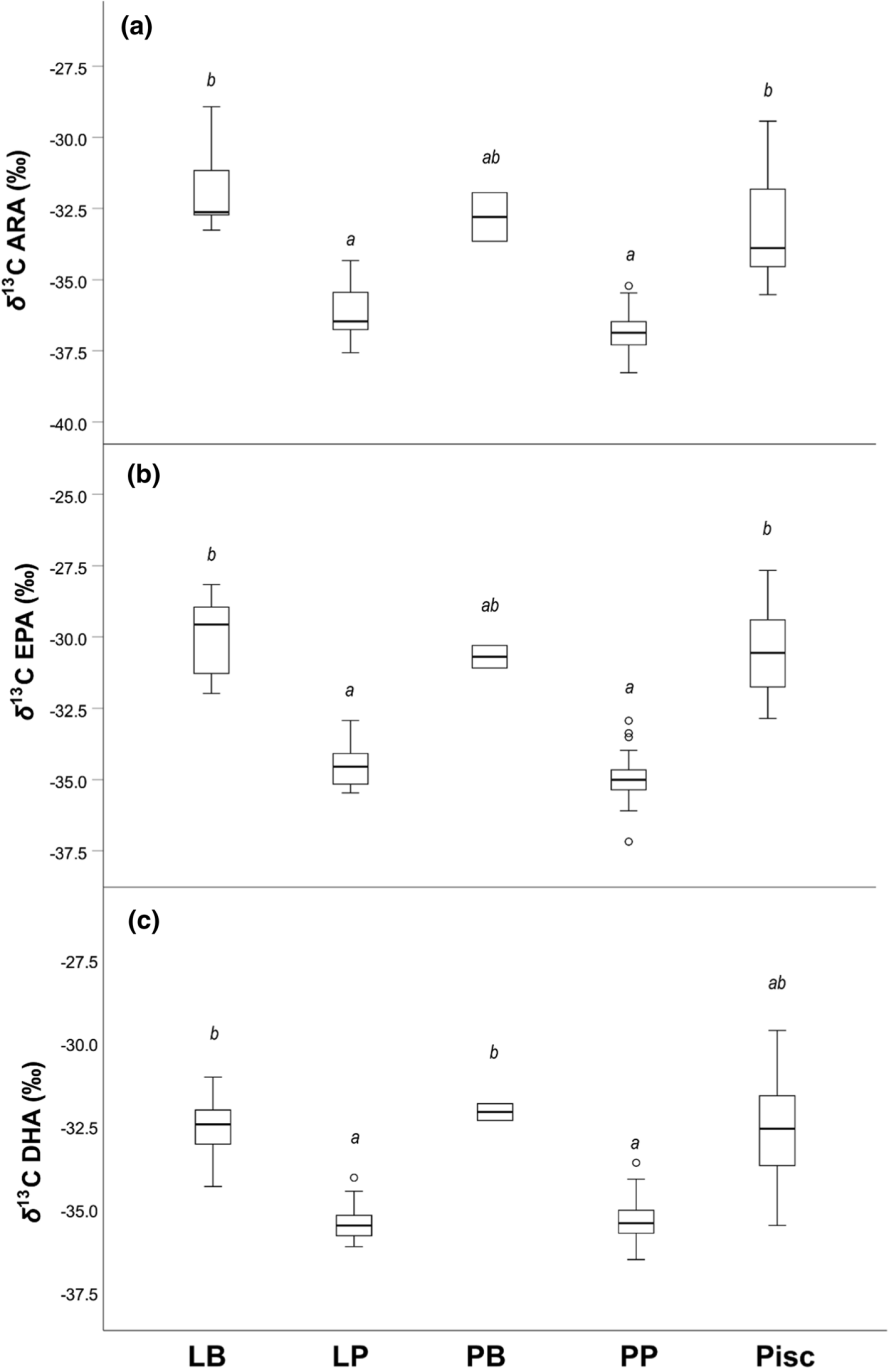
HUFA δ^13^C of the different feeding types of perch. Boxplot are depicted for δ^13^C in **a** ARA; **b** EPA; **c** DHA in littoral benthivorous (LB), littoral planktivorous (LP), pelagic benthivorous (PB), pelagic planktivorous (PP), and littoral piscivorous perch (Pisc). Feeding types with the same letter are not significantly different (Bonferroni-adjusted Dunn’s pairwise comparisons). Boxplots depict median, 25th and 75th percentile, and whiskers extend to maximum and minimum values, except for outliers (> 1.5 times box height, represented by dots)

Kruskal–Wallis test of *δ*^13^C of all three HUFAs explored indicated significant differences between the feeding types (ARA: *H* = 47.238, *df* = 4, *P* < 0.001; EPA: *H* = 67.552, *df* = 4, *P* < 0.001; DHA: *H* = 57.653, *df* = 4, *P* < 0.001). For all three HUFAs, littoral planktivorous and pelagic planktivorous perch were significantly depleted in ^13^C, whereas the other three feeding types showed similar signatures (Fig. 3).

## Discussion

Our study suggests that HUFA levels in perch muscle reflected their composition in their diet with respect to EPA, but there was a mismatch between diet and consumer levels with respect to ARA and DHA. This pattern was particularly pronounced in DHA, where realized values of DHA in perch muscle tissue exceeded the theoretical values on average by a factor of five. Thus, our results are in-line with a recent meta-analysis of feeding studies in fish and aquatic invertebrates suggesting that DHA is retained even when supplied in high levels (Jardine et al. 2020). Due to the important role of DHA in fish metabolism (e.g. Parrish 2009), this may not come as a surprise. DHA has very specific molecular functions and is particularly involved in fish reproduction (Izquierdo et al. 2001; Scharnweber et al. 2020; Tocher 2003). DHA may be obtained directly through resource consumption, but our study suggests that bioconversion might also be a relevant route to obtain sufficient amounts of this HUFA in freshwater fish.

Using CSIA to identify the origin of the carbon atoms used to build up HUFAs, we found indications that perch are capable of bioconverting fatty acids. Generally, perch individuals that were assigned to feeding on benthic resources were characterized by higher values of δ^13^C in their HUFAs (i.e. being enriched in ^13^C). In contrast, perch individuals assigned to feeding on pelagic resources showed lower values of δ^13^C in their HUFAs (i.e. being depleted in ^13^C). Thus, *δ*^13^C values of HUFAs in perch reflected the signatures of their respective resources. For example, DHA signatures of littoral benthivorous perch that feed on ^13^C-enriched benthic invertebrates were enriched in ^13^C, indicating littoral origin of the DHA carbon. Although benthic invertebrates show very low proportions of DHA, perch consumers have nonetheless DHA proportions similar to perch feeding on DHA-rich resources (Online Resource 2). Thus, our results suggest that perch can alter their HUFA levels by transforming from shorter chain precursors. This would be accomplished via bioconversion during internal transformations, where LIN serves as a precursor for ARA, while ALA serves as precursor for EPA that can further be transformed into DHA (Bell et al. 2009).

When comparing the isotopic signatures of EPA and DHA, a general pattern of lower values in DHA becomes apparent. Irrespective of diet or habitat of perch, DHA signatures were significantly more depleted compared to EPA signatures (Mann–Whitney *U *Test; *P* ≤ 0.001, average across all feeding types ± SD: EPA − 33.6 ± 2.2‰; DHA: − 34.6 ± 1.5‰). This can be explained by the preferential addition of the lighter ^12^C during biosynthesis (DeNiro et al. 1977, 1978). Little is known about fractionation factors of these processes. Fujibayashi et al. (2016) reported a trophic discrimination of -2.6 ‰ between dietary ALA and observed DHA signatures in the zebrafish (*Danio rerio*) during feeding trials, but variation might occur for different diets (Bec et al. 2011; Gladyshev et al. 2016). Nonetheless, modeling results from Bec et al. (2011) suggested that if endmembers are substantially isotopically distinct (i.e. in the range of 10 ‰, which is applicable for our data), CSIA data are reliable to draw conclusions about trophic interactions and transfer pathways of biomolecules. Certainly, discrimination factors for perch feeding types are needed to give precise estimates for the extent of HUFA bioconversion in this species. As an alternative explanation for the observed pattern of high-DHA proportions in benthic perch, individuals could obtain some amount of their DHA from pelagic resources despite the minor contribution to the biomass production of the fish. Such process of selective retention has been described, e.g. for a species of mullet (*Liza saliens*) (Koussoroplis et al. 2010) and salmonids (Heissenberger et al. 2010). Furthermore, piscivorous perch could also obtain a significant amount of their DHA from bioconversion occurring at lower trophic levels, i.e. their fish prey. This could explain the rather similar signatures of benthivorous and piscivorous perch.

It is widely assumed that rates of trophic upgrading for HUFAs are low and most of the vast amount of HUFAs need to be acquired by dietary intake (Bell et al. 2009). However, the modeling attempt of Sawyer et al. (2016), estimated that the dominant uptake pathway for EPA in Yellow perch [*Perca flavescens* (Mitchill 1814)], a close relative to the Eurasian perch, would be ingestion, but up to 87% of the Yellow perch’s DHA would be obtained via internal conversion. Unfortunately, we cannot estimate transformation rates from our data, and further experimental studies are needed to determine the specific physiological processes involved to regulate HUFA levels in natural perch feeding types, but more generally also on the uptake and transfer of trophic biomarkers in organisms across the whole animal kingdom (Galloway et al. 2020).

In this study, we measured the proportions of HUFAs from muscle tissue only. However, fatty acids can be selectively transferred and catabolized from different tissues and organs (Henderson et al. 1984; Schwalme et al. 1993; Tocher 2003). For example, gonadal development and overwintering are energy costly processes that lead to a mobilization from tissues, including muscles (Keva et al. 2019; Muir et al. 2014). To fully understand the extent of internal regulation, fatty acid levels need to be estimated also from other important lipid storage tissue (e.g. gonads, liver, eyes, and brains), to obtain a full overview and balance of HUFA integration.

Due to the aforementioned uncertainties, our results may not be interpreted as direct empirical evidence of bioconversion, but certainly, it is a strong indication for this process. Previous studies have only considered the capability of freshwater fish species reared in aquaculture and when fed on rather artificial diet (Henrotte et al. 2011; Murray et al. 2015; Xu et al. 2002), but to our knowledge, this study is the first report for this process occurring on the natural scale and for wild populations of fish.

Henrotte et al. (2011) showed that the bioconversion efficiency in perch in aquaculture depends on ontogeny, with juvenile fish being more efficient in transforming the ALA precursor into EPA and DHA. As DHA is required for growth, brain, and eye development (Tocher 2010), such high enzymatic efficiencies are especially beneficial to growth during early life stages. In this study, we did not test for age or ontogenetically linked HUFA level variations in perch. However, Chaguaceda et al. (2020) found ontogenetic processes, represented by changes in total length, to be responsible for about a third of the variation in overall fatty acid composition of 38 fatty acids analyzed. Similarly, Lane et al. (2011) observed a difference in fatty acid signatures by age in Atlantic herring [*Clupea harengus* (L.)]. During the life-history of a fish, the physiological requirements for a specific fatty acid change (Tocher 2010). For example, ARA and DHA are needed for the formation of gonads and a greater supply is needed at the onset of maturity (Tocher 2003). Therefore, the predominant influence of ontogeny on the energetic requirement of an animal (Werner et al. 1984) may be highly influential also on the degree of internal transformation of HUFA in natural populations of freshwater fish.

The different feeding types responded differently to the specific HUFA levels of the respective resource. For example, perch individuals specialized on benthic resources that are generally low in DHA did not show corresponding lower proportions of DHA in their muscle tissue (Online Resource 2). Instead, they had the highest accumulation factor indicating higher proportions in muscle tissues than the ones found in their resources. This result contrasts to the findings of Happel et al. (2015) and Chavarie et al. (2016), who reported a clear association of higher DHA levels and pelagic feeding in Yellow Perch and Lake Trout, respectively. Furthermore, while factors of ARA and DHA were larger than 1, factors of EPA were close to 1, indicating a close match between realized and theoretical assumed proportions. Nonetheless, when investigating the factors of the specific feeding types, a striking mismatch became again apparent. While the factor for individuals feeding on benthic resources was well above 1, it was substantially lower for planktivorous and piscivorous perch. The general difference between feeding types of perch in this mismatch between HUFA levels and resources as well as the degree of homeostasis is interesting. For example, individual specialization in freshwater perch is not based on assortative mating, and differences between the perch types are not genetically fixed, but mainly due to phenotypic plasticity (Faulks et al. 2015; Marklund et al. 2019; Olsson et al. 2005; Svanbäck et al. 2006). Therefore, we can assume that physiological adaptations to cope with resources that have different HUFA levels are also rather flexible and plastic. So far, we know little about the potential costs for consumers and the trade-offs involved in dealing with qualitatively different resources. Stream macroinvertebrates are considered as ‘selective retainers’ instead of simple ‘collectors’ of dietary fatty acids (Guo et al. 2016). Furthermore, genes that are responsible for fatty acid bioconversion and even de novo synthesis of HUFA were recently found to be widespread across the animal kingdom (Kabeya et al. 2018; Monroig et al. 2018). To understand the degree of selective retention but also the immediate ecological and even evolutionary consequences of keeping up the enzymatic machinery involved in the bioconversion of HUFAs opens a new and exciting avenue for future research.

## Supplementary Information

Below is the link to the electronic supplementary material.

Supplementary file1 (DOCX 136 KB)
