# Supplementary material for: Fatty acid accumulation in feeding types of a natural freshwater fish population
Source: Oecologia. 2021 Apr 25;196(1):53–63. doi: 10.1007/s00442-021-04913-y (PMC8139920; doi:10.1007/s00442-021-04913-y)

**Fatty acid accumulation in feeding types of a natural freshwater fish population**

Scharnweber, Kristin^*^, Chaguaceda, Fernando & Eklöv, Peter

Uppsala University, Department of Ecology and Genetics; Limnology, Uppsala, Sweden

^*^ **Corresponding author:** Kristin.Scharnweber@ebc.uu.se, Phone: +46 18 471 2796, Fax: +46 18 471 6310, ORCID-ID: 0000-0003-2858-5947

**Electronic Supplemental Material**

**Online Resource 1: Stable isotopes of resources and perch feeding types.** Sample sizes (*N*), averages and standard deviations for δ^13^C and δ^15^N of a) resources used in the mixing models and b) the different feeding types: littoral benthivorous (LB), littoral planktivorous (LP), pelagic benthivorous (PB), pelagic planktivorous (PP), and littoral piscivorous perch (Pisc)

|  | *N* | δ^13^C (‰) | δ^15^N (‰) |
| --- | --- | --- | --- |
| a) Resources |  |  |  |
| Cladocera | 2 | -28.6 ± 0.4 | 5.0 ± 0.4 |
| Copepoda | 2 | -31.4 ± 0.4 | 9.4 ± 0.6 |
| Benthic Macroinvertebrates | 4 | -22.4 ± 0.9 | 6.5 ± 0.7 |
| Fish | 6 | -25.2 ± 1.0 | 10.6 ± 1.6 |
| b) Perch feeding types |  |  |  |
| LB | 12 | -24.5 ± 0.6 | 10.3 ± 0.7 |
| LP | 28 | -27.2 ± 0.5 | 10.5 ± 0.8 |
| PB | 2 | -24.8 ± 0.3 | 9.6 ± 0.7 |
| PP | 55 | -27.5 ± 0.4 | 11.1 ± 0.4 |
| Pisc | 16 | -25.0 ± 1.2 | 11.9 ± 0.6 |

**Online Resource 2: Proportions of HUFAs in muscle tissue of perch.** Boxplots are depicted for the different feeding types: littoral benthivorous (LB), littoral planktivorous (LP), pelagic benthivorous (PB), pelagic planktivorous (PP), and littoral piscivorous perch (Pisc). a) ARA; b) EPA; c) DHA. Feeding types with the same letter are not significantly different (Bonferroni-adjusted Dunn´s pairwise comparisons)


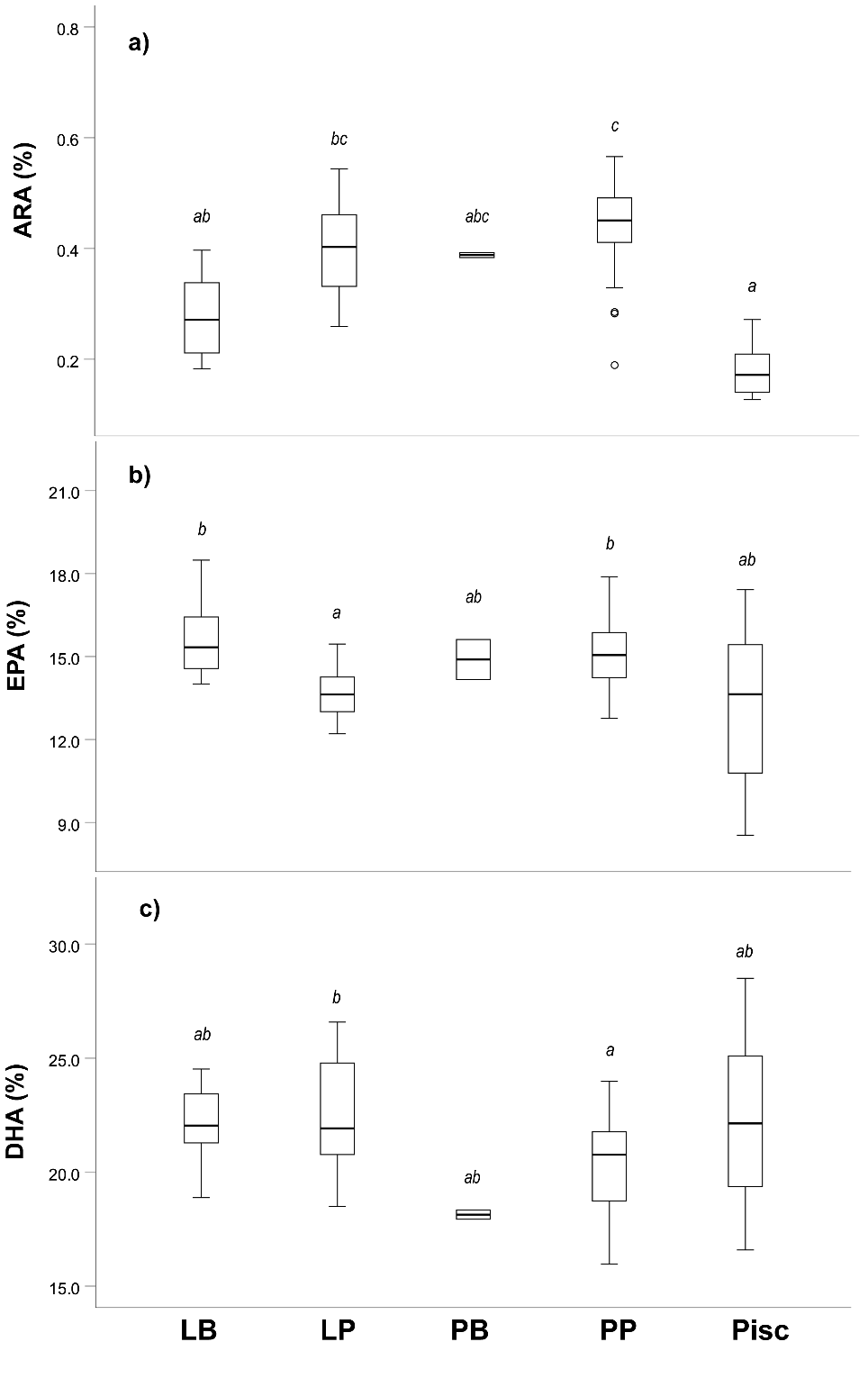

Supplement: Supplementary file 1 — Supplementary file1 (DOCX 136 KB) [file 442_2021_4913_MOESM1_ESM.docx]
